# Physiological Properties and Genome Structure of the Hyperthermophilic Filamentous Phage **φ**OH3 Which Infects *Thermus thermophilus* HB8

**DOI:** 10.3389/fmicb.2016.00050

**Published:** 2016-02-23

**Authors:** Yuko Nagayoshi, Kenta Kumagae, Kazuki Mori, Kosuke Tashiro, Ayano Nakamura, Yasuhiro Fujino, Yasuaki Hiromasa, Takeo Iwamoto, Satoru Kuhara, Toshihisa Ohshima, Katsumi Doi

**Affiliations:** ^1^Faculty of Agriculture, Institute of Genetic Resources, Kyushu UniversityFukuoka, Japan; ^2^Department of Bioscience and Biotechnology, Faculty of Agriculture, Kyushu UniversityFukuoka, Japan; ^3^Faculty of Arts and Science, Kyushu UniversityFukuoka, Japan; ^4^Faculty of Agriculture, Attached Promotive Center for International Education and Research of Agriculture, Kyushu UniversityFukuoka, Japan; ^5^Core Research Facilities, Research Center for Medical Sciences, Jikei University School of MedicineTokyo, Japan; ^6^Department of Biomedical Engineering, Faculty of Engineering, Osaka Institute of TechnologyOsaka, Japan

**Keywords:** hyperthermophilic phage, *Thermus thermophilus*, filamentous phage, *Inoviridae*, replicative form

## Abstract

A filamentous bacteriophage, φOH3, was isolated from hot spring sediment in Obama hot spring in Japan with the hyperthermophilic bacterium *Thermus thermophilus* HB8 as its host. Phage φOH3, which was classified into the *Inoviridae* family, consists of a flexible filamentous particle 830 nm long and 8 nm wide. φOH3 was stable at temperatures ranging from 70 to 90°C and at pHs ranging from 6 to 9. A one-step growth curve of the phage showed a 60-min latent period beginning immediately postinfection, followed by intracellular virus particle production during the subsequent 40 min. The released virion number of φOH3 was 109. During the latent period, both single stranded DNA (ssDNA) and the replicative form (RF) of phage DNA were multiplied from min 40 onward. During the release period, the copy numbers of both ssDNA and RF DNA increased sharply. The size of the φOH3 genome is 5688 bp, and eight putative open reading frames (ORFs) were annotated. These ORFs were encoded on the plus strand of RF DNA and showed no significant homology with any known phage genes, except ORF 5, which showed 60% identity with the gene VIII product of the *Thermus* filamentous phage PH75. All the ORFs were similar to predicted genes annotated in the *Thermus aquaticus* Y51MC23 and *Meiothermus timidus* DSM 17022 genomes at the amino acid sequence level. This is the first report of the whole genome structure and DNA multiplication of a filamentous *T. thermophilus* phage within its host cell.

## Introduction

Thermophilic phages or viruses play extraordinarily important roles in the processes of evolution, biogeochemistry, ecology, and genetic exchange in extreme environments (Prangishvili et al., 2006). Among these phages, those that infect *Thermus* species have been extensively studied (Liu et al., 2009), and complete genome sequences have been reported for myoviruses YS40 (Naryshkina et al., 2006) and TMA (Tamakoshi et al., 2011); siphoviruses P23-45, P74-26 (Minakhin et al., 2008), TSP4 (Lin et al., 2010), and φIN93 (Matsushita and Yanase, 2009); and tectivirus P23-77 (Jalasvuori et al., 2009). As far as we know, however, no genome information has been reported for a filamentous phage that infects *Thermus* species.

Filamentous phages belong to the *Inoviridae* family. As reported by Ackermann (Ackermann, 2007), phages belonging to this family are far fewer in number than tailed phages. The inovirus virions contain a circular, positive sense, single-stranded DNA (ssDNA) genome within a helical array composed of thousands of copies of the major capsid protein. As a result of this structural arrangement, inoviruses are flexible filaments about 7 nm in diameter. Inoviruses infect both gram-negative and gram-positive bacteria (Day, 2012), but are unusual among bacteriophages in that they do not lyse their host cells when new phage particles are produced. Instead, new virions are packaged at the cell surface and extruded (Rakonjac et al., 1999; Marvin et al., 2014). These virions contain ssDNA that typically enters new hosts via pili on the cell surface (Stassen et al., 1994). Once inside the host, inoviruses persist in a circular, double-stranded replicative form (RF); alternatively, they can integrate into the host chromosome through the actions of phage-encoded transposases (Kawai et al., 2005) and host-encoded XerC/D (Huber and Waldor, 2002; Hassan et al., 2010), which normally resolves chromosome dimers. Production of new phage ssDNA can then proceed via rolling-circle replication from the RF. The genomes of inoviruses are composed of modules that encode proteins involved in genome replication, virion structure and assembly, and regulation (Campos et al., 2010). Like many other phages, inoviruses can undergo extensive recombination, often picking up new genes in the process, so that they may act as important vectors for gene transfer among hosts (Davis and Waldor, 2003; Faruque et al., 2005).

Phage PH75 is the only reported inovirus isolated from *T. thermophiles* HB8 (Yu et al., 1996). Although structural analysis of its coat proteins has been carried out (Pederson et al., 2001; Overman et al., 2004; Tsuboi et al., 2005), to our knowledge there is no reported genome information for phage PH75. Thirty-six species of inovirus listed by International Committee on Taxonomy of Viruses (ICTV) have been isolated from enterobacteria and the *Pseudomonas, Vibrio*, and *Xanthomonas* species. Among them, the genomes of the enterobacteria phages fd (Beck et al., 1978), f1 (Hill and Petersen, 1982), Ike (Peeters et al., 1985), and M13 (van Wezenbeek et al., 1980); the *Propionibacterium* phage B5 (Chopin et al., 2002); *Pseudomonas* phages Pf1 (Hill et al., 1991) and Pf3 (Luiten et al., 1985); and *Vibrio* phages VCYφ (Xue et al., 2012); and *Yersinia* phage Ypfφ (Derbise and Carniel, 2014) have all been completely sequenced. Because all of these filamentous phages infect mesophilic bacteria, comparison of the genome of a hyperthermophilic *Thermus* inovirus and the aforementioned mesophilic inoviruses may provide important insight into the diversity, evolution, and ecology of bacteriophages. In particular, it may shed light on the features that confer thermostability to the phage proteins encoded by its genomic DNA.

In this report, we characterize phage φOH3, a filamentous phage infecting *T. thermophiles* HB8, including its temperature, pH and salt tolerances, as well as its host range, one-step growth curve and replicative behavior in host cells. We also report the structure of the φOH3 genome the first example from a *Thermus* filamentous phage.

## Materials and methods

### Geothermal water sample

The hot spring water samples from which φOH3 was isolated were collected from the Obama hot spring, Nagasaki, Japan (32°43′25″N, 130°12′50″E, 75°C).

### Bacterial strains, media, and growth conditions

*Thermus thermophilus* HB8 (Oshima and Imahori, 1971) was used as the host strain for phage φOH3 in this study. *T. thermophilus* strains AT-62 (Saiki et al., 1972), HB27 (Oshima and Imahori, 1971), TMY (Fujino et al., 2008), and Fiji3 A.1 (Saul et al., 1993) as well as *Thermus aquaticus* YT-1 (Brock and Freeze, 1969), *Meiothermus ruber* strain 21 (Loginova and Egorova, 1975), and *Geobacillus kaustophilus* NBRC 102445 (Priest et al., 1988) were also used as indicators. *T. thermophilus* strains were cultivated in HB8 broth (Sakaki and Oshima, 1975) at 70°C with shaking at 180 rpm. Castenholz medium (Nold and Ward, 1995) was used for cultivation of *T. aquaticus* YT-1 and *M. ruber* strain 21 at 70 and 55°C, respectively. *G. kaustophilus* NBRC 102445 was cultivated in Nutrient broth (Nissui Pharmaceutical, Japan) at 55°C with shaking at 180 rpm.

Plasmid pUC18 was used for cloning and analysis of the nucleotide sequence of the φOH3 genome. *Escherichia coli* DH5α was grown in Luria-Bertani (LB) medium at 37°C (Sambrook and Russell, 2001). When required, 50 μg/ml ampicillin was added. SM buffer [100 mM NaCl, 8 mM MgSO_4_ 7H_2_O, 50 mM Tris-HCl (pH 7.5), and 0.002% (w/v) gelatin] was used for storage and dilution of phage particles (Sambrook and Russell, 2001).

### Isolation and purification of phage

A geothermal water sample was added to an equal volume of HB8 broth for an enrichment culture. After cultivation (70°C, 180 rpm, 2 days), the culture was centrifuged (6000 × g, 10 min, 4°C) to remove bacterial cells and debris, after which and the supernatant was passed through a nitrocellulose filter with 0.45 μm pores (Advantec, Japan). The filtrate was then added to an equal amount of double strength HB8 broth supplemented with 10 mM CaCl_2_ and inoculated with a log-phase host culture (OD_660_ = 0.4). The phage was assayed using the soft agar overlap technique (Adams et al., 1959). After overnight incubation (70°C), typical plaques were suspended in SM buffer and purified through 10 rounds of single-plaque isolation.

### Transmission electron microscopy

Phage morphology was determined using transmission electron microscopy to observe negatively stained preparations (Luo et al., 2012). One drop containing approximately 10^12^ PFU/ml freshly prepared purified phage solution was applied to the surface of a Formver-coated grid (200 mesh copper grid), negatively stained with 2% (wt/vol.) phosphotungstic acid (pH 7.2), and then examined using an Hitachi H-7500 transmission electron microscope operated at 80 kV (Hitachi High-Technologies Corp., Japan).

### Host range determination

The host range of the phage was investigated using the spot test (Shirling and Speer, 1967) against five *T. thermophilus* strains (HB8, HB27, AT62, TMY, and Fiji3 A.1) as well as *T. aquaticus* YT-1, *M. ruber* 21, and *G. kaustophilus*. Incubation temperatures were set with optimum temperatures for each strain.

### Heat, pH, and saline stability

All plaque assays were performed using a 0.8% TM agar overlay on 2.0% TM agar. Plaque development occurred within 1 day, and assays were conducted in triplicate. Thermal, pH and salt stabilities were tested as described previously (Lin et al., 2011) with modification. Briefly, to examine of thermal stability, phage stocks (1.0 × 10^7^ PFU/ml in SM buffer) were incubated separately for 60 min at 50, 60, 80, and 90°C. The phage titer was then evaluated using the double-layer method with early log phase *T. thermophilus* HB8 cells at a multiplicity of infection (MOI) of 1. For pH stability, 1.0 × 10^7^ PFU/ml phage solution was added into modified TM broth (pH values were adjusted from 3 to 11) and then incubated for 24 h at 20~70°C. To asses salt stability, sodium chloride was dissolved to 0, 0.1, 0.5, 1.0, or 3.0 M with phage lysates (1.0 × 10^7^ PFU/ml), and the mixture was incubated for 24 h at 20~70°C. The resultant phage solutions were diluted to 1.0 × 10^4^ PFU with TM broth and then used to infect HB8 cells at a MOI of 0.1. The plates were then incubated overnight at 70°C before examination for the presence of plaques.

### One-step growth curve

A one-step growth curve was constructed as previously described (Pajunen et al., 2000) with some modifications. An early-exponential-phase culture (10 ml) of *T. thermophilus* HB8 (OD_660_ = 0.4) was harvested by centrifugation and resuspended in 1 ml of fresh TM broth (10^8^ CFU/ml). φOH3 was added to the HB8 suspension at a MOI of 10 and allowed to adsorb for 15 min at 70°C. Thereafter, the mixture was washed with an equal volume of fresh TM broth. This manipulation was repeated three times to remove any free phage particles. After centrifugation, the pelleted cells were resuspended in 100 ml of fresh TM broth and incubated for 2 h at 70°C while shaking. During the incubation, samples were taken at 10-min intervals and immediately centrifuged, after which the supernatants were plated on TM agar to determine the phage titer. Number of released virion was calculated as the ratio of the final count of liberated phage particles to the initial number of infected bacterial cells during the latent period like as burst size of lytic phage (Adams et al., 1959).

### Extraction and purification of phage genomic DNA

φOH3 genomic DNA was obtained from its host cells using a Cica Geneus Plasmid Prep Kit (KANTO CHEMICAL, Japan) with continuous extraction every 10 min after infection. Extracted DNA samples were separated using 0.8% agarose gel electrophoresis, and then ssDNA and RF DNA were visualized using acridine orange staining (Mayor and Hill, 1961; McMaster and Carmichael, 1977). Determination of the copy number of ssDNA and RF DNA within the φOH3 genome was done using ImageJ ver 1.48 (http://rsb.info.nih.gov/ij/) by comparing the intensities of their fluorescent signals. The peak areas of both the ssDNA and RF signal were quantified and used to calculate the copy number. To purify RF DNA, the plasmid-like genomes were isolated from 5-ml samples of *T. thermophilus* HB8 after cultivation for 80 min. The φOH3 genomic DNA was then separated on 1.0% agarose gels, and the bands corresponding to the RF DNA were excised and purified using a MinElute Gel Extraction Kit (QIAGEN GmbH, Germany).

### Genome characterization

RF DNA was digested using *Hin*d III, *Kpn* I, or *Pst* I, after which the resultant φOH3 RF fragments were inserted into separate pUC18 vectors. Each recombinant plasmid was then used to transform *E. coli* DH5α competent cells. Nucleotide sequencing of the plasmids was accomplished through primer walking using a BigDye Terminator v3.1 Cycle Sequencing Kit (Life Technologies, CA, USA). Sequences were determined using an Applied Biosysytems Gene Analyzer 3130xl (Life Technologies). The sequence of the φOH3 genome was also obtained by pyrosequencing performed with a 454 Genome Sequencer FLX system (Roche, Schweiz) as described previously (Doi et al., 2013). Open reading frames (ORFs) were predicted using a combination of MiGAP and GENETYX ver. 15 (GENETYX, Japan). Translated ORFs were analyzed through blastp, PsiBlast, rpsblast, and hhpred searches.

### DNA blot analysis

To confirm that ORFs were located on the plus strand, DNA blot analysis was carried out as described by Liu et al. (2012). Non-denatured DNA samples were separated on a 1.0% agarose gel and then transferred to a nylon membrane (Amersham Hybond-N+; GE Healthcare, UK) through capillary action. The probes corresponding to each strand of RF DNA in the blotting experiments were prepared using DIG Northern Starter Kit (Roche). The DNA probe used in the Southern blotting experiments was a 294-bp DNA fragment derived from ORF2, which was prepared by PCR. The primer set used for the PCR was derived from the coding region of ORF2 and is as follows: orf2-f (5′-ATGAAGGTTTTGGTTCTAGGAGT-3′) and orf2-r (5′-TCACGCCTTGACCTCCT-3′). The amplified ORF2 gene was inserted into the pTA2 vector (TOYOBO, Japan), and the resultant plasmid was digested with *Hin*d III or *Pst* I, after which the linearized plasmid served as the template for RNA transcription catalyzed using T3 or T7 RNA polymerase. Hybridization of each RNA product, RNA probe-1and -2, to the phage DNA and detection were carried out according to the manufacturer's instructions.

### Structural protein identification by mass spectroscopy

To analyze φOH3 virion proteins, phage solution (10^10^ pfu/ml) was concentrated with Amicon Ultra-15 filter (NMWL 50000; Merck Millipore, Germany) and then purified using DISMIC membrane filter (0.45 mm; ADVANTEC, Japan). The purified phage particles were mixed with lysis buffer (62.5 mM Tris-HCl, pH 6.8, containing 5% 2-mercaptoethanol, 2% sodium dodecyl sulfate, 10% glycerol, and 0.01% bromophenol blue) and boiled for 10 min. The prepared proteins were then separated by electrophoresis on precast Tricine-SDS 15% polyacrylamide gels (e-PAGEL E-T/R15S; ATTO Corporation, Japan).

### In-gel digestion

Excised gel pieces were de-stained with acetonitrile solution. After the gel plugs were dried, 400 ng of sequencing-grade trypsin (Trypsin Gold, Promega, Madison, WI) in 20 μl of 25 mM ammonium bicarbonate were added and incubated for 12 h at 37°C. Selected gel plugs were also treated with chymotrypsin. Digested peptides were recovered from the gel plugs using 50 μl of 50% acetonitrile in 5% formic acid (FA) for 30 min at 25°C. The extracted peptides were concentrated in a speed vacuum concentrator and added to 20 μl of 5% acetonitrile in 0.1% FA.

### Protein identification

A Nano-HPLC system (nanoADVANCE, Bruker Michrom., Billerica, MA) was used to identify proteins automatically using a micro-column switching device coupled to an autosampler and a nanogradient generator. Peptide solution (5 μl) was loaded onto a C18 reversed-phase capillary column (100 μm ID × 30 cm, Zaplous α X Pep C18: AMR, Tokyo, Japan) in conjunction with a Magic C18AQ trapping column (300 μm ID × 10 mm, Bruker). The peptides were separated using a nanoflow linear acetonitrile gradient of buffer A (0.1% FA) and buffer B (0.1% FA, 99.9% acetonitrile), going from 5 to 45% buffer B over 50 min at a flow rate of 500 nl/min. The column was then washed in 95% buffer B for 5 min. Hystar 3.2 system-control software (Bruker Daltonics Inc., Billerica, MA) was used to control the entire process. The eluted peptides were ionized through a CaptiveSpray source (Bruker Daltonics) and introduced into a Maxis 3G Q-TOF mass spectrometer (Bruker Daltonics Inc., Billerica, MA) set up in a data-dependent MS/MS mode to acquire full scans (m/z acquisition range from 50 to 2200 Da). The four most intense peaks in any full scan were selected as precursor ions and fragmented using collision energy. MS/MS spectra were interpreted and peak lists were generated using DataAnalysis 4.1 and Biotools 3.2.

### Bioinformatics

The filtered data were searched on the Mascot 2.2 server (Matrix Science) using the NCBInr (NCBI 20150627) custom genome sequence and custom expected protein databases for φOH3. The custom φOH3 genome sequence database was created by dividing every 2000 sequences from the φOH3 genome sequence (LC035386). Fixed modification was set on cysteine with carbamidomethylation. Variable modification was based on methionine with oxidation and asparagine/glutamine with deamidation. Maximum missed cleavage was set to 2 and limited to trypsin cleavage sites. Precursor mass tolerance (MS) and fragment mass tolerance (MS/MS) were set to 100 ppm and ± 0.6 Da, respectively. Positive protein identifications using a threshold of 0.05 were used. Peptides scoring <20 were automatically rejected, ensuring all protein identifications were based on the reliable peptide identifications.

### Nucleotide sequence accession number

The genome sequence of φOH3 has been deposited in GenBank with accession number LC035386.

## Results

### Isolation and morphology of φOH3

**Figure 1:**
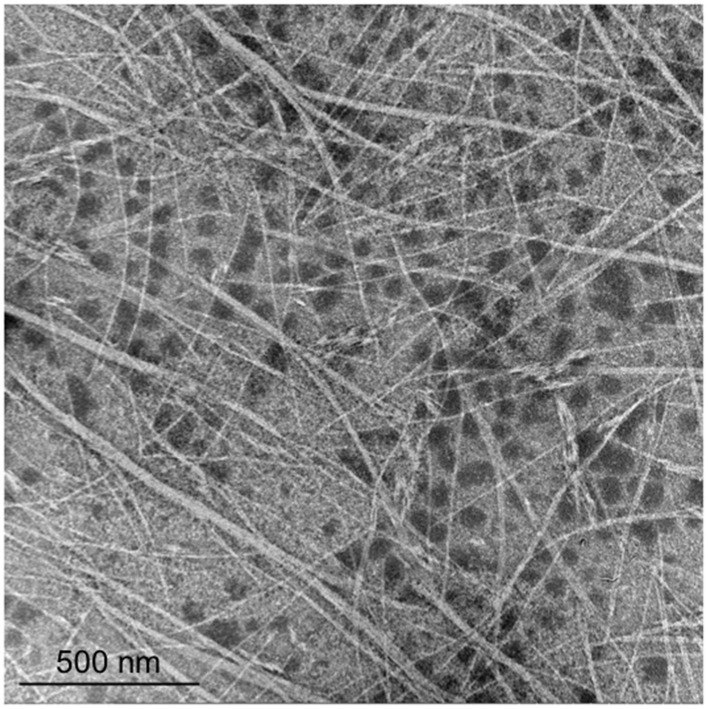
**Morphology of phage φOH3**. Shown are transmission electron micrographs of inovirus φOH3 negatively stained with 2% (wt/vol) potassium phosphotungstate (pH 7.0). The scale bar is 500 nm.

Large numbers of plaques were formed from the enrichment culture of a geothermal water sample using *T. thermophilus* HB8 as the host. One of them, φOH3, formed turbid plaques 0.5–1.1 mm in diameter on the lawn of *T. thermophilus* HB8 after incubation for 12 h at 70°C. Transmission electron microscopy and negative staining revealed the morphology of φOH3 to consist of flexible filaments, approximately 8 nm wide and 830 nm long (Figure 1). On that basis, phage φOH3 was classified as a member of the family *Inoviridae*.

### Host range of φOH3

The infectivity of phage φOH3 was examined using various thermophilic bacteria, including *T. thermophilus* HB8, *T. thermophilus* HB27, *T. thermophilus* AT62, *T. thermophilus* TMY, *T. thermophilus* Fiji3 A.1, *T. aquaticus* YT-1, *M. ruber* strain 21, and *G. kaustophilus* NBRC 102445. Phage φOH3 formed an inhibitory zone only on *T. thermophilus* HB8.

### Heat, pH, and salt tolerance

**Figure 2:**
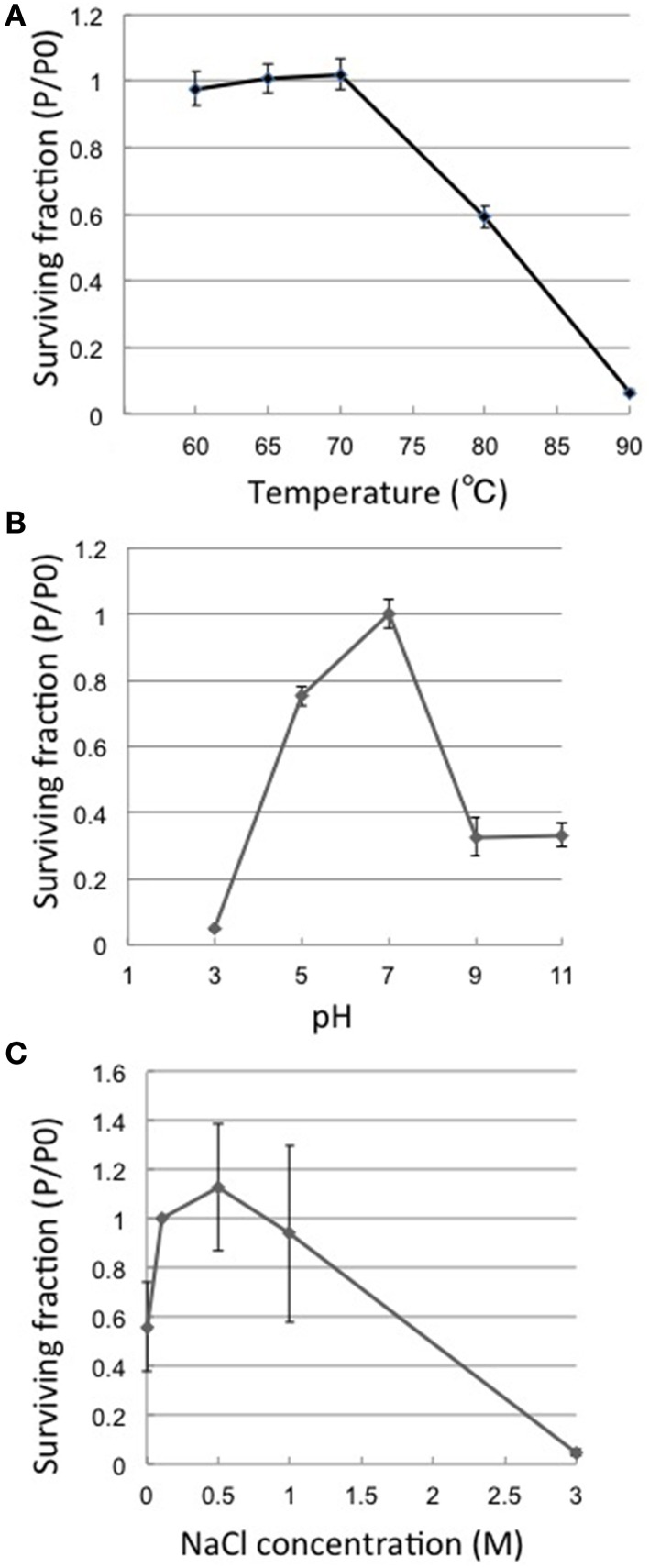
**Heat, pH and salt stability of φOH3**. Surviving fractions (P/P0) are plotted, where P0 is the φOH3 initial titer, and P is the mean titer from triplicate assays after incubation for 1 h at the indicated temperature **(A)**, 24 h at the indicated pH **(B)**, or 24 h at the indicated NaCl concentration **(C)**.

Thermostability assays showed that phage φOH3 was most stable at 70°C. After incubation for 1 h at 80 or 90°C, the φOH3 survival fraction dramatically decreased to 59.2 and 6.5%, respectively (Figure 2A), and the phage was completely inactivated at 100°C (data not shown). φOH3 was most stable at pH 7.0 and was sensitive to both acidic and alkali pHs (Figure 2B). At lower or higher pHs, phage survival decreased substantially. For example, when the pH was lower than 3.0 or higher than 9.0, the phage survival fraction was reduced to 4 and 32.7%, respectively.

Phage φOH3 was stable in NaCl at concentrations ranging from 0 to 1 M. (Figure 2C), and was most stable in SM buffer, which contains 0.5 M NaCl. In solution containing 3M NaCl, the phage survival fraction was only 1.1%. In the complete absence of NaCl, the survival fraction was reduced to 60%.

### Phage particle propagation and genome replication

**Figure 3:**
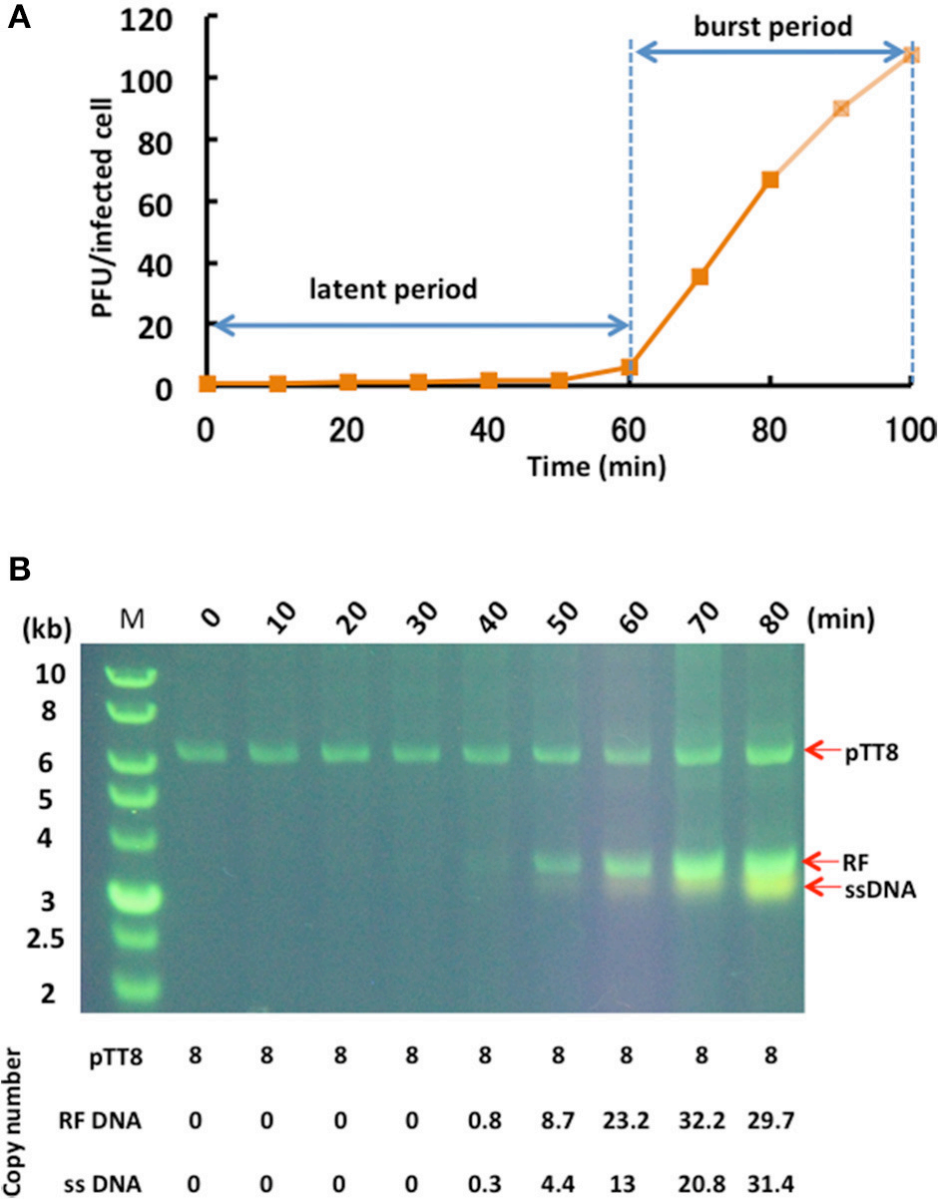
**Propagation of φOH3 in ***T. thermophilus*** HB8. (A)** One-step growth curve of φOH3. **(B)** Agarose gel electrophoresis of phage φOH3 genomic DNA. The φOH3 genome was extracted with alkaline lysis after the indicated cultivation periods. Double stranded RF DNA and ssDNA were distinguished by acrydine orange staining. Corresponding estimated copy numbers of pTT8 (Takayama et al., 2004), RF DNA, and ssDNA of φOH3 are shown under the gels.

The one-step growth curve for phage φOH3 showed the latent period and release period to be 60 and 40 min, respectively (Figure 3A). The phage adsorption fraction was 90.0%, and 109 phage particles were released from each infected cell, on average. Agarose gel electrophoresis showed that the φOH3 genome was present in HB8 cells as both RF DNA and ssDNA (Figure 3B). While changes in the copy number of RF DNA were detected 40–80 min after infection, changes in ssDNA copy number increased sharply after 50 min. Overall, the intracellular DNA concentrations increased in proportion to the duration of cultivation. Because the copy number of the cryptic plasmid pTT8 (9328 bp) of *T. thermophilu*s HB8 remained constant at 8 throughout the latent and release periods (Hishinuma et al., 1978; Takayama et al., 2004), we were able to use it as an internal standard and determine that the copy numbers of the RF DNA and ssDNA comprising the φOH3 genome were roughly 30 and 16, respectively, 80 min after infection.

### Phage genome analysis

**Figure 4:**
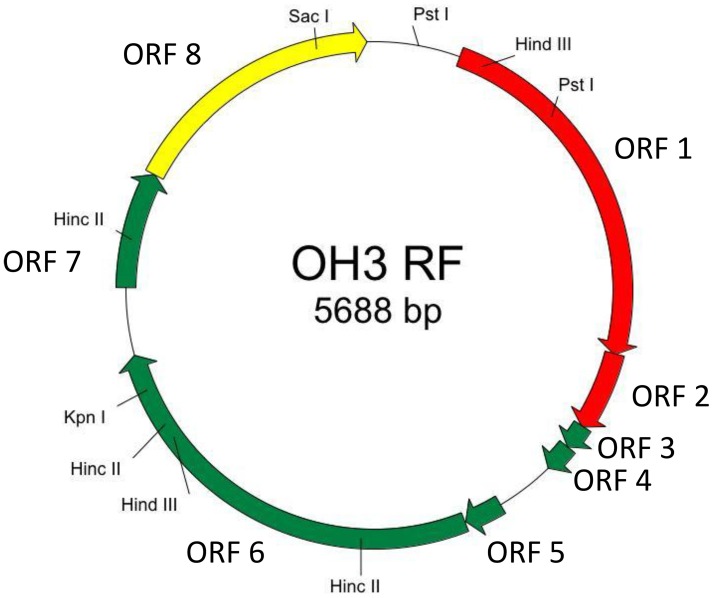
**Genetic map of φOH3 RF DNA**. Predicted ORFs are indicated by arrows. Arrow colors show putative gene functions as follows: replication, red; structure, green; assembly, yellow. Since the conserved domain of Zot was detected in the ORF8 product, ORF8 might function for phage assembly.

**Figure 5:**
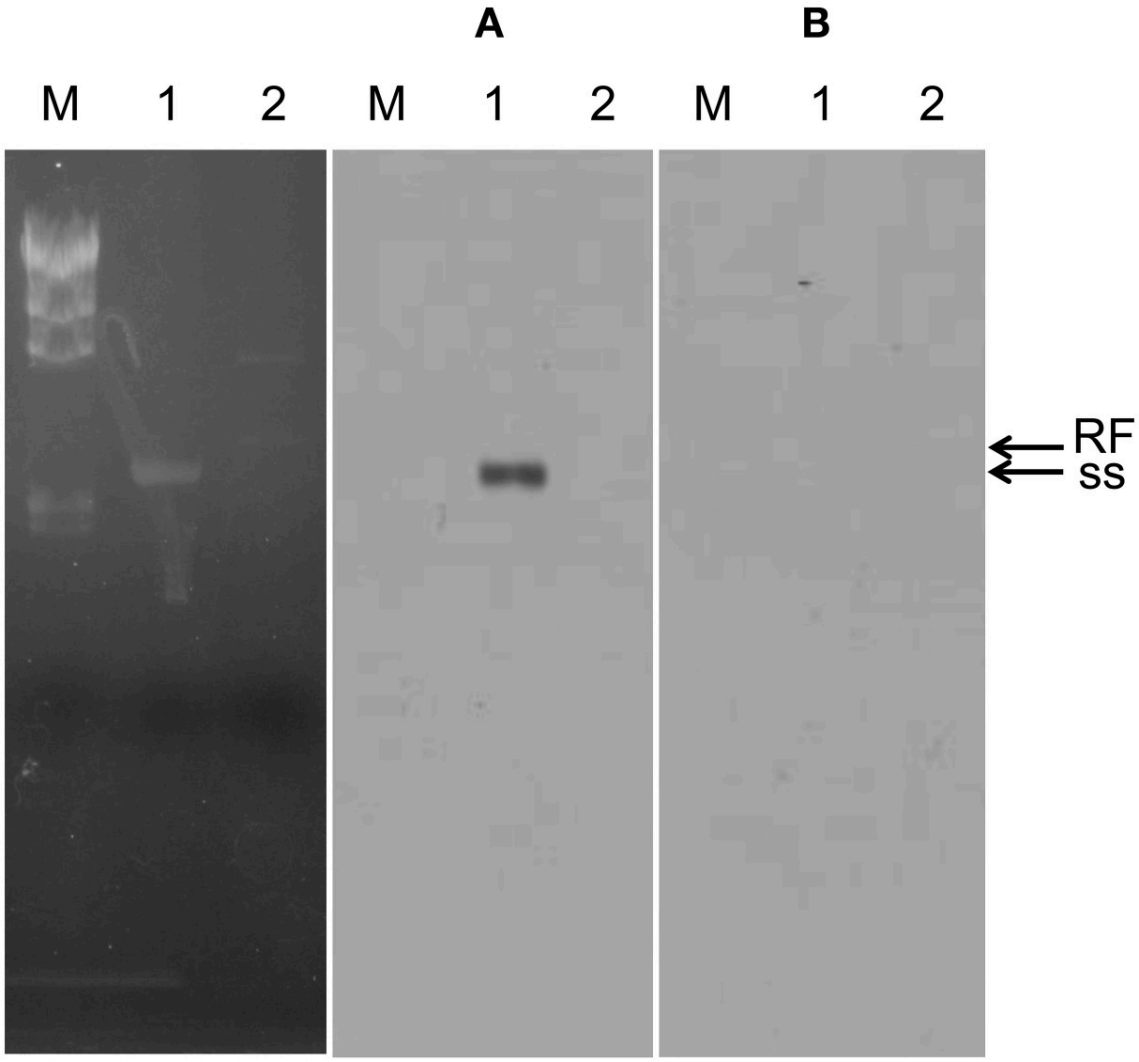
**Southern blot confirming the plus strand of phage φOH3**. Lanes: M, lambda -*Hin*d III marker; 1, φOH3 ssDNA; 2, φOH3 RF DNA. **(A)** RNA probe-1, which was transcribed by T7 RNA polymerase (antisense strand of ORF2). **(B)** RNA probe-2, which were transcribed by T3 RNA polymerase (sense strand of ORF2). Bands corresponding to ss DNA or RF DNA are indicted with arrows.

**Figure 6:**
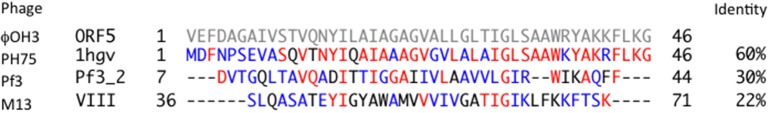
**Comparison of the amino acid sequences of ORF5 product (φOH3), 1hgv (PH75), Pf3_2 (Pf3), and g8p (M13)**. Identical and similar amino acid residues are indicated in red and blue, respectively. Hyphens represent gaps in the alignment.

The genome of φOH3 consists of 5688 base pairs (bp) with a G+C content of 58.03%, which is significantly lower than the 69.52% of its host strain, *T. thermophilus* HB8 (NCBI RefSeq accession number: NC_006461; Figure 4). Southern hybridization using oligoprobes designed from each strand of the RF sequence showed that the φOH3 genome was single stranded (Figure 5). Using MiGAP pipeline, six ORFs longer than 100 bp were predicted from the sequence of the plus strand, which was revealed by Southern hybridization. In addition, two small ORFs (<100 bp) were predicted based on comparative genome analysis (Table 1). These eight putative ORFs had no significant homology with any phage genes registered in databases at the nucleotide sequence level. On the other hand, the amino acid sequence of the ORF V product showed 60% identity with P8, the major coat protein of phage PH75 (Pederson et al., 2001; Figure 6), while the amino acid sequences of the other putative ORF products showed similarities to putative uncharacterized proteins encoded in the *T. aquaticus* Y51MC23, *Meiothermus timidus* DSM 17022 and *Thermus* sp. genomes (GenBank accession numbers: ABVK00000000, ARDL00000000, and JTJB00000000; Table 1). Based on comparisons with the genomes of these strains, ORFs 3 and 4 were presumed to be situated between ORFs 2 and 5. A conserved domain of Zonula occludens toxin (Zot) (Koonin, 1992) was detected in the N-terminal region (A.A. 1–197) of the ORF8 product. Although gene localization within φOH3 is similar to that in M13, fd, B5, and Pf3, four other filamentous phages, there are no genes homologous to gene I encoding the assembly protein in the φOH3 genome.

**Table 1:** **Protein-encoding region predicted in the φOH3 genome and their relationship to amino acid sequences from the databases**.

**ORF no**.	**Position (5′ → 3′)**	**Corresponding gene in M13**	**Putative protein function**	**Homologous protein**	**Identity**	***e*-value**	**Accession no**.
1	319–1659	II	RF replication Endonuclease	Hypothetical protein (*Thermus aquaticus* Y51MC23)	54%	9e^−146^	WP_003044556.1
				Hypothetical protein (*Thermus* sp. 2.9)	55%	2e^−145^	WP_039459567.1
2	1656–1949	V	Single-strand DNA binding protein	Hypothetical protein (*Thermus aquaticus* Y51MC23)	51%	4e^−21^	WP_003044553.1
				Hypothetical protein (*Meiothermus timidus* DSM 17022)	41%	9e^−15^	WP_018467932.1
3	1946–2038	VII	Minor coat protein	Hypothetical protein (*Thermus aquaticus* Y51MC23)	62%	5e^−04^	EED10729.1
4	2039–2143	IX	Minor coat protein	Hypothetical protein (*Thermus aquaticus* Y51MC23)	68%	4e^−06^	EED10728.1
5	2363–2503	VIII	Major coat protein	Hypothetical protein (*Meiothermus timidus* DSM 17022)	64%	0.016	WP_018467931.1
				Hypothetical protein (*Thermus* sp. 2.9)	65%	0.063	WP_039459577.1
				Hypothetical protein (*Thermus aquaticus* Y51MC23)	61%	1.4	EED10727.1
6	2507–4027	III	Adsorption protein	Hypothetical protein (*Thermus aquaticus* Y51MC23)	50%	6e^−136^	EED10726.1
7	4277–4705	VI	Minor coat protein	Hypothetical protein (*Thermus* sp. 2.9)	61%	6e^−43^	WP_039459592.1
				Hypothetical protein (*Thermus filiformis* ATCC 43280)	59%	5e^−36^	WP_038066844.1
				Hypothetical protein (*Thermus aquaticus* Y51MC23)	52%	4e^−29^	WP_003046233.1
8	4705–5661	IV	Morphogenesis	Hypothetical protein (*Thermus* sp. 2.9)	86%	5e^−177^	WP_039459711.1
				Hypothetical protein (*Thermus aquaticus* Y51MC23)	81%	4e^−150^	WP_003046231.1
				Hypothetical protein (*Meiothermus timidus* DSM 17022)	76%	1e^−149^	WP_018467929.1

### Protein analysis of φOH3

**Figure 7:**
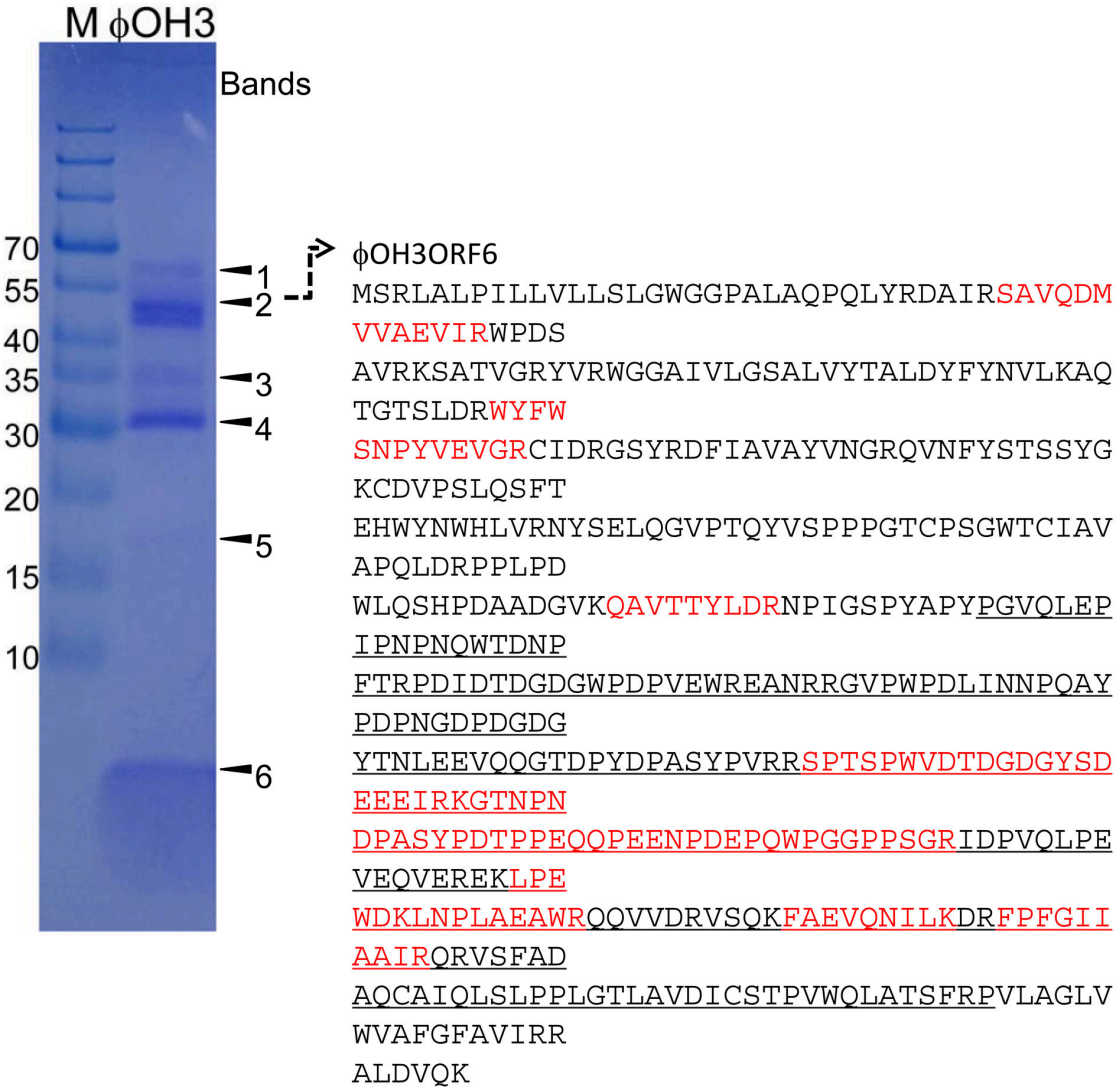
**SDS-PAGE of proteins from purified φOH3 virions staining with Coomassie brilliant blue R250**. The numbers indicate the bands excised for mass spectrometric analysis (Supplemental Tables 1, 2). The left lane contains protein molecular mass markers (kDa). Red colored and underlined characters indicate matched peptides and domain of DNA translocase FtsK (PRK10263).

Purified phage particles were analyzed using SDS-PAGE (Figure 7). Six major bands with estimated molecular masses of 60, 55, 35, 30, 17, and 5 kDa, respectively, were found. The bands were then subjected to bioinformatics analysis using the bacteria sequence databases of NCBInr, which showed that all the protein bands included proteins from *Thermus thermophiles* (Supplemental Table 1). With a custom φOH3 database, analysis using six-frame translation showed that the peptides from the 55 kDa band matched the sequence region of the φOH3ORF 6 sequence (Supplemental Table 2 and Figure 7). No other significant matches were found with six-frame translation of the DNA database. Nine unique peptides were matched against the φOH3ORF 6 sequence, giving 27% total sequence coverage (matched 136 A.A./total 506 A.A.). With a Blastp search, we found that the φOH3ORF 6 sequence includes the DNA translocase FtsK domain structure (PRK10263) and shows 50% identity with the hypothetical protein TaqDRAFT_4286 (*Thermus aquaticus* Y51MC23, gb|EED10726.1|). The peptides from 5 kDa band were also covered from the sequence of φOH3ORF5 (Supplemental Table 2 and Figure 7). Initial proteomics analysis using trypsin digestion failed to identify the protein. Since the cleavage sites of trypsin in φOH3ORF5 sequence are only located at the C-terminal region, possible tryptic fragments are expected to be 38 amino acids or longer. Proteomics analysis, especially high throughput measurement, is not efficient at obtaining sequence information from peptides that are more than 30 residues long. Sequences of three uniques chymotryptic bands were detected. However, these mascot scores were insignificant.

## Discussion

We have shown that the filamentous phage φOH3 infects the hyperthermophilic bacterium *T. thermophilus* HB8. To date, several kinds of *Thermus* phages belonging to the *Mioviridae, Siphoviridae, Tectiviridae*, and *Inoviridae* families have been isolated and characterized (Yu et al., 2006). φOH3 appears to be a *Thermus* filamentous phage belonging to the family *Inoviridae*. PH75 is another filamentous phage that infects *T. thermophilus* HB8. Although the physiological characteristics of pVIII, the major coat protein in PH75, have been investigated using Raman and UV-resonance Raman spectroscopy (Overman et al., 2004; Tsuboi et al., 2005), there is presently no information on the PH75 genome sequence in any database, as far as we know. Detailed characteristics of this phage also have never been published. Consequently, biological characterization and genome analysis of a thermophilic filamentous phage could provide new information for virology, physiology, nanotechnology, and so on.

Thermostability assays showed that φOH3 was most stable at 70°C, which is similar to the optimal temperature for φYS40, but is higher than that for phages TSP4 (most stable at 60°C), MMP17 (most stable at 55–60°C), and GVE1 (most stable at 60°C), whose hosts are *T. thermophilus* HB8 (φOH3 and φYS40), *Thermus* TC4 (TSP4), *Meiothermus* TG17 (MMP17), and *Geobacillus* sp. E26323 (GVE1), respectively. The high thermostability of φOH3 may reflect the growth temperature of the host bacterium *T. thermophilus* HB8 rather than its filamentous morphology. The pH stability of φOH3 resembled that of MMP17 but was less than that of TSP4. In addition, Sakaki and Oshima reported that φYS40 is sensitive to high concentrations of salt (NaCl) (1975). Like φYS40, φOH3 was inactivated in the presence of 3 M NaCl. The dominant ions in the Obama hot spring waters from which φOH3 was isolated are Cl and Na, with concentrations ranging from 0.06 to 0.37 M and 0.04 to 0.31 M, respectively (Saibi and Ehara, 2010). From these results, it appears that the temperature, pH and NaCl sensitivity of phage φOH3 reflect its taxonomic position and/or geographic origin.

In the life cycle of Inoviruses, injection of the phage ssDNA through bacterial membranes into the cytoplasm is carried out after pilus-mediated adsorption of the phage onto its host cell. Thereafter, the host polymerase converts the ssDNA into covalently closed double-stranded DNA, which is the replicative form. In φOH3, it takes the host polymerase at least 40 min to convert the ssDNA to RF DNA (Figure 3B). Finally, newly generated plus ssDNA strands are converted into new RF DNA molecules. It is noteworthy that the release period was initiated simultaneously with ssDNA replication, that continuous replication of both ssDNA and RF DNA is observed throughout the release period, and that plasmid pTT8 replication was unaffected by the phage DNA replication.

**Figure 8:**
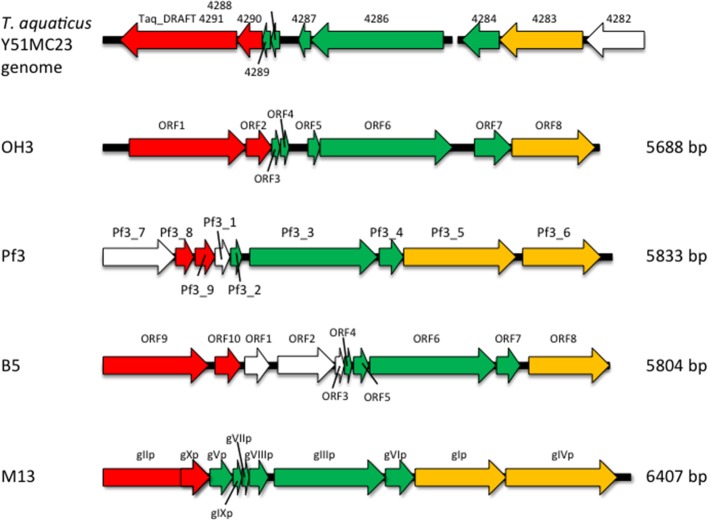
**Comparison of the genome organization of phage φOH3 with that of the phage Pf3, B5, M13, and ***T. aquaticus*** Y51MC23 genomes**. Open reading frames with similar functions are indicated using the same color scheme used in Figure 4. White arrows show regions encoding hypothetical proteins with unknown functions.

Nucleotide analysis of the φOH3 genome showed it to be 5688 bp in size, which is approximately the same as other filamentous phages such as Pf3 (5833 bp), B5 (5804 bp), and M13 (6407 bp; Figure 8). Although BLASTn revealed no homologous genes within the φOH3 genome, BLASTx revealed significant homology with predicted genes in the genomes of *T. aquaticus* Y51MC23 and *M. timidus* DSM 17022 (Figure 8). It thus appears that φOH3-like phages are able to infect *T. aquaticus* or *Meiothermus* species and that these phage genomes are integrated into their host chromosomes. No genes encoding an integrase or transposase are present in the φOH3 genome. Because the homologous genes are located on discrete contigs in each strain, the φOH3-related genes may be defective phages or traces of phage integration. Phage φOH3 could not infect either *T. aquaticus* or *Meiothermus* species (data not shown), and no hybridized bands were detected in the *T. aquaticus* YT-1 or *M. timidus* DSM 17022 genome using φOH3 DNA as a probe (data not shown), though ssDNA or RF DNA from φOH3 or a homolog could be transformed to these species with their natural competency. The amino acid sequence of the ORF 5 product shows high similarity (60%) to 1 hgv of phage PH75, but low similarity (<30%) to major coat proteins from other filamentous phages, such as Pf3_2 from Pf3 or protein VIII from M13. It seems likely the structural differentiation arose for the hyperthermostability of the φOH3 and PH75 virions (Tsuboi et al., 2005; Russel and Model, 2006).

Because nearly all the ORFs in the φOH3 genome are annotated as similar to other predicted genes with unknown function, we applied ESI-MS/MS and nano LC-MS/MS to identify the phage structural proteins. Because of difficulty for purification with ultracentrifugation, we selected to concentrate the phage particles using MS analysis enabled experimental examination of structural proteins and confirmed the genome-based gene predictions (Lavigne et al., 2009). Peptide sequences of the ORF 6 product could be detected with ESI-MS/MS. We cloned all the predicted ORFs in *E. coli* and examined the properties and localization of the gene products. These results will be reported elsewhere in the near future. Further comparative study of the thermophilic filamentous phage φOH3 will shed light not only on the mechanism underlying the thermostability of the proteins but also on mechanisms of gene evolution and transfer in a geothermal environment.

## Author contributions

The work presented here was carried out in collaboration between all authors. KD and TO defined the research theme. KD, YN, AN, and YF designed methods and experiments, carried out the virological experiments, analyzed the data, interpreted the results, and wrote the paper. KM, KT, and SK co-designed and analyzed DNA sequencing experiments, and co-worked on associated data collection and their interpretation. YH and TI co-designed protein analysis and ESI-MS/MS experiments, discussed analyses, interpretation, and presentation. All authors have contributed to, seen and approved the manuscript.

## Funding

This work was partly funded by JSPS KAKENHI No. 24658083, 26292182, and JST A-STEP No. 15650473.

### Conflict of interest statement

The authors declare that the research was conducted in the absence of any commercial or financial relationships that could be construed as a potential conflict of interest.
