# Supplementary material for: Physiological Properties and Genome Structure of the Hyperthermophilic Filamentous Phage φOH3 Which Infects Thermus thermophilus HB8
Source: Front Microbiol. 2016 Feb 23;7:50. doi: 10.3389/fmicb.2016.00050 (PMC4763002; doi:10.3389/fmicb.2016.00050)
Supplement: Supplementary file 1 [file DataSheet1.pdf]

Supplemental Table 1. Bacterial protein identification identified by ESI-MS/MS.
* Protein search in bacterial sequence databalse of NCBInr

Band No.	gi number	protein description	prot_score	protein MW	Unique peptides matched	peptide charge	miss cleavage	peptide score	pep_seq	modification
Band 1										
	gi|499548193	hypothetical protein [Thermus thermophilus]	353	100832	9					
						2	0	55	SSVTASDLPISVR	
						2	0	51	FLVSASEEGSGWSAR	
						2	0	30	DGGDDDALETSDDVVR	
						2	0	28	VSAISYEEHAALLYK	
						2	0	32	FSFQGIGESYSGQPVK	
						2	0	28	ADLTYTTAGAYAISVNK	
						3	1	43	QKQPTTDLLPITGFGYVR	
						2	0	38	GCETQNVTELDFVMTDADGIAK	Oxidation (M)
						3	0	47	VDLCDISPATCAAAINDTDGSGVYLQPK	
Band 2										
	gi|499548193	hypothetical protein [Thermus thermophilus]	307	100832	8					
						2	0	29	SSVTASDLPISVR	
						2	0	57	FLVSASEEGSGWSAR	
						3	0	31	VFFYNITHLYLR	
						2	0	40	DGGDDDALETSDDVVR	
						3	0	30	VSAISYEEHAALLYK	
						2	0	50	FSFQGIGESYSGQPVK	
						2	0	31	GCETQNVTELDFVMTDADGIAK	Oxidation (M)
						3	0	38	VDLCDISPATCAAAINDTDGSGVYLQPK	
	gi|499487356	ABC transporter substrate-binding protein [Thermus thermophilus]	133	48146	4					
						2	0	50	LALYQQYWAAR	
						2	0	25	IAPQGVTSYAEEEAR	
						2	0	27	NWPYAYALGQAEGSPIR	
						3	0	33	YNQVSEAIWTEVHSVLTGR	
Band 3										
	gi|499548193	hypothetical protein [Thermus thermophilus]	310	100832	7					
						2	0	37	SSVTASDLPISVR	
						2	0	57	FLVSASEEGSGWSAR	
						2	0	45	DGGDDDALETSDDVVR	
						3	0	50	VSAISYEEHAALLYK	
						2	0	42	FSFQGIGESYSGQPVK	
						2	0	49	GCETQNVTELDFVMTDADGIAK	Oxidation (M)
						3	0	30	VDLCDISPATCAAAINDTDGSGVYLQPK	
	gi|499548190	hypothetical protein [Thermus thermophilus]	200	36238	4					
						2	0	43	VLYQFLDTDFGDR	
						3	1	42	GGVKVDGVPGVGTAGTYVLAEPR	
						3	0	47	HYGTGSAGCLGQGPVSFQAVLYPDGR	
						2	2	67	KQGIDNLKELITNYK	Deamidated (NQ)
Band 4										
	gi|499548193	hypothetical protein [Thermus thermophilus]	357	100832	8					
						2	0	33	YELVGGDVSK	
						2	0	67	SSVTASDLPISVR	
						2	0	70	FLVSASEEGSGWSAR	
						2	0	28	DGGDDDALETSDDVVR	
						2	0	43	VSAISYEEHAALLYK	
						2	0	41	FSFQGIGESYSGQPVK	
						2	0	42	GCETQNVTELDFVMTDADGIAK	Oxidation (M)
						2	0	32	VDLCDISPATCAAAINDTDGSGVYLQPK	
	gi|480311946	iron ABC transporter substrate-binding protein [Thermus thermophilus]	184	36952	4					
						2	0	42	AYPSNPAMLDAIR	
						2	0	52	DGDAGNLALVTGAGLLK	
						2	0	50	YSTDAQILAALQEEGSR	
						2	0	41	SPADLFWANTAGALGQASAK	
	gi|499547028	phosphate ABC transporter substrate-binding protein [Thermus thermophilus]	96	35954	3					
						2	0	35	ISVAFSGTGAGFQK	
						2	0	38	STGYVELPDEAYR	
						2	0	23	TGSFFTDLPPGTPLAK	
Band 5		None								

Supplemental Table 2. Phage protein identification identified by ESI-MS/MS.
* Protein search in predicted expressed protein databalse of OH3

Band No.	gi number	protein description	prot_score	protein MW	Unique peptides matched	peptide charge	miss cleavage	peptide score	pep_seq	modification
Band 1		OH3ORF4	291	56881	9					
						2	0	30	FAEVQNILK	
						2	0	41	QAVTTYLDR	
						2	0	39	LNPLAEAWR	
						2	0	33	FPFGIIAAIR	
						2	0	73	SAVQDMVVAEVIR	
						2	0	40	WYFWSNPYVEVGR	
						3	1	22	LPEWDKLNPLAEAWR	
						3	1	24	SPTSPWVDTDGDGYSDEEEIRK	
						3	0	25	GTNPNDPASYPDTPPEQQPEENPDEPQWPGGPPSGR	

No other maches found
